# Supplementary material for: Resolving the full spectrum of human genome variation using Linked-Reads
Source: Genome Res. 2019 Apr;29(4):635–45. doi: 10.1101/gr.234443.118 (PMC6442396; doi:10.1101/gr.234443.118)
Supplement: Supplemental Material [file supp_gr.234443.118_Supplemental_longranger-2.2.zip › longranger-2.2.tar.bz2!/lr2.2-public/lib/stan/cmdstan/stan/doc/api/html/math_2functions_2as__bool_8hpp_source.html]

Stan: src/stan/math/functions/as\_bool.hpp Source File


|  |  |
| --- | --- |
|  | Stan  2.5.0  probability, sampling & optimization |


- Main Page
- Related Pages
- Namespaces
- Classes
- Files

- File List
- File Members

All Classes Namespaces Files Functions Variables Typedefs Enumerations Enumerator Friends Macros Pages

- src
- stan
- math
- functions

as\_bool.hpp

Go to the documentation of this file.

1 #ifndef STAN\_\_MATH\_\_FUNCTIONS\_\_AS\_BOOL\_HPP

2 #define STAN\_\_MATH\_\_FUNCTIONS\_\_AS\_BOOL\_HPP

3

4 namespace stan {

5  namespace math {

6

13  template <typename T>

14  inline bool as\_bool(const T x) {

15  return x != 0;

16  }

17

18  }

19 }

20

21 #endif

stan::math::as\_bool

bool as\_bool(const T x)

Return 1 if the argument is unequal to zero and 0 otherwise.

**Definition:** as\_bool.hpp:14


---

[ Stan Home Page ]

*© 2011–2014,
Stan Development Team.*
